# Profiling of Sediment Microbial Community in Dongting Lake before and after Impoundment of the Three Gorges Dam

**DOI:** 10.3390/ijerph13060617

**Published:** 2016-06-21

**Authors:** Wei Huang, Xia Jiang

**Affiliations:** State Key Laboratory of Environmental Criteria and Risk Assessment, Chinese Research Academy of Environmental Sciences, Beijing 100012, China; yixinghd6@163.com

**Keywords:** bacterial community, sediment, high throughput sequencing, water period, impoundment

## Abstract

The sediment microbial community in downstream-linked lakes can be affected by the operation of large-scale water conservancy projects. The present study determined Illumina reads (16S rRNA gene amplicons) to analyze and compare the bacterial communities from sediments in Dongting Lake (China) before and after impoundment of the Three Gorges Dam (TGD), the largest hydroelectric project in the world. Bacterial communities in sediment samples in Dongting Lake before impoundment of the TGD (the high water period) had a higher diversity than after impoundment of the TGD (the low water period). The most abundant phylum in the sediment samples was *Proteobacteria* (36.4%–51.5%), and this result was due to the significant abundance of *Betaproteobacteria* and *Deltaproteobacteria* in the sediment samples before impoundment of the TGD and the abundance of *Gammaproteobacteria* in the sediment samples after impoundment of the TGD. In addition, bacterial sequences of the sediment samples are also affiliated with *Acidobacteria* (11.0% on average), *Chloroflexi* (10.9% on average), *Bacteroidetes* (6.7% on average), and *Nitrospirae* (5.1% on average). Variations in the composition of the bacterial community within some sediment samples from the river estuary into Dongting Lake were related to the pH values. The bacterial community in the samples from the three lake districts of Dongting Lake before and after impoundment of the TGD was linked to the nutrient concentration.

## 1. Introduction

The operation of large-scale water conservancy projects can change the natural hydrological cycles and sediment translocation processes in downstream linked lakes [1,2]. The damming of rivers has had a significant global impact on natural water resources [3], as impoundment of dams can affect the water environments, *i.e.*, physical, chemical, and biological characteristics, as well as the hydrology of neighboring lakes or rivers.

Yangtze River is the largest river in China (6300 km); it is the fifth largest river in the world in terms of fresh water discharge (9.8 × 10^11^ m^3^/year), and the fourth largest in solid discharge (4.86 × 10^8^ t/year) [4]. The Three Gorges Dam (TGD) is on the lower section of the upper reaches of the Yangtze River, and is the largest hydroelectric project in the world. The construction of the TGD began in 1993, and was completed in 2009, with some exceptions. The project has played a significant role in controlling frequent catastrophic floods downstream, generating hydropower (18,200 MW), water storage (12.4 billion m^3^, 2003), and improving navigation at the upper reaches of the Yangtze River. However, the TGD affects ecosystems, known as TGD’s upstream and downstream effects, which stem from inundation, flow manipulation, and fragmentation [5]. Dongting Lake is located in the middle and lower reaches of the Yangtze River. It is one of the largest freshwater lakes in China, and plays a significant role in regulating the amount of water entering the Yangtze River. The inflow from the Yangtze River carries an average of 4 million MT of sediment load per year [6]. In recent years, the impoundment of the TGD has been implemented at the end of September. Before impoundment of the TGD, the water level in Dongting Lake was high and the lake was in its high water period, with the average water level reaching 27.36 m. After impoundment of the TGD, the water input from Yangtze River decreased and the lake entered its low water period with the average water level of 21.51 m. The phenomenon of dry–rewet cycles occurred and the sediments at some positions exposed from the water when the lake was in low water period.

As a distinct realm in aquatic ecosystems, sediments have higher biomass and microbe taxon richness than the bodies of water [7]. Prokaryotes, especially bacteria, play a dominant role in fresh water lake sediments, promoting nutrient recycling and decomposition of organic or inorganic compounds [8,9]. Therefore, a shift in bacterial communities may be one of the most sensitive indicators of environmental changes in the lake [10]. The impoundment of the TGD has a significant effect on the Yangtze River, and it also affects the sediment in Dongting Lake because of its location. In recent years, the sediment in Dongting Lake has been influenced by the operation of TGD, such as the sediment deposition, the unsaturated sediment carrying capacity in the downstream-linked riverway, and riverbed sediment erosions occurring downstream after the impoundment of TGD, thereby causing siltation [11,12,13]. In addition, microbial community changes in sediment also occur due to the impoundment of TGD. A few studies have addressed the microbial community composition affected by the TGD along the Yangtze River using more course-level culture-independent methods such as denaturing gradient gel electrophoresis (DGGE) and clone libraries [14,15,16]. As one of the most significant microbes, bacteria in the sediment in Dongting Lake are crucial to the entire lake’s ecosystem.

The relationship between the Dongting Lake and the TGD has been studied by scholars from different perspectives, and most of the researchers focused on the early dry season in the downstream lakes, which is caused by TGD and nutrient characteristics, translocation, or sediment discharge [17,18]. The potential effects of TGD impoundment on Dongting Lake have been investigated in previous studies that focused on prediction, verification, and analysis of soil properties and biomass in the early low-water period [18,19,20]. However, few studies have been conducted on the effects of the impoundment of the TGD on the sediment bacterial community in Dongting Lake, and traditional techniques present inherent limitations in bacterial community studies. For example, when employing fingerprinting techniques, such as DGGE and phospholipid fatty acid analysis (PLFA), precise, accurate, and comprehensive description of microbial communities are difficult to obtain [21,22,23]. Thus, the objective of the present study was to compare the sediment bacterial communities before and after impoundment of the TGD using Illumina Miseq. This study may provide new insights into the bacterial communities in the sediment of Dongting Lake.

## 2. Materials and Methods

### 2.1. Site Description and Sediment Sampling

**Figure 1:**
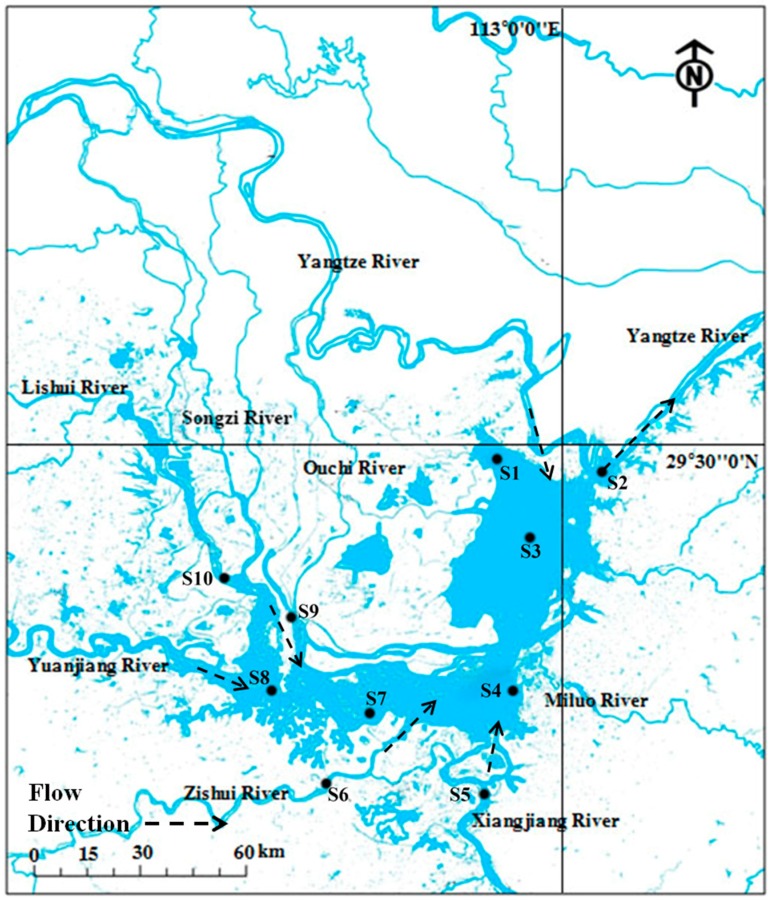
Sampling site at the study area.

Dongting Lake, which is the second largest freshwater lake in China, is located in Hunan Province (E 111°40’–113°10’, N 28°38’–29°45’). Dongting Lake is composed of a series of lakes and has three major lake districts, namely the eastern, southern, and western districts. The sample positions are shown in Figure 1. Sediment 1 (S1) and Sediment 3 (S3) were from East Lake. Sediment 4 (S4) and Sediment 7 (S7) were from south lake. Sediment 8 (S8) was from west lake. Sediment 2 (S2) was from the outlet of Dongting Lake and the estuary of the Yangtze River. Sediment 5, 6, 9, and 10 (S5, S6, S9, and S10) were from the river estuary into Dongting Lake. Ten composite surface sediment samples (each *N = 5*) were collected before (in July 2015, high water period,) and after (in October 2015, low water period) the impoundment of the Three Gorges Dam, and these samples were assigned as HS and LS, respectively. The weight of sample collected was sufficient for DNA extraction and analysis of physicochemical parameters. Sediment samples were put into sealed plastic bags and stored in a portable ice box, then transferred to the lab as quickly as possible and stored at −80 °C before analysis.

The sediment samples were divided into four groups. The samples of Group A (HS1, HS3, HS4, HS7 and HS8) and Group B (HS2, HS5, HS6, HS9 and HS10) were collected from the three lake districts of Dongting Lake and the river estuary into Dongting Lake during the high water period, respectively. The collection sites of the samples from Group C (LS1, LS3, LS4, LS7 and LS8) and Group D (LS2, LS5, LS6, LS9 and LS10) were the same as Groups A and B, respectively, and the samples were collected during the low water period.

### 2.2. Analysis of Physicochemical Parameters

The total phosphorus (TP) of the sediments was measured by using standardized methods and tests, and total nitrogen (TN) of the sediments was obtained as ammonium by colorimetry after the sample was digested with alkaline potassium persulfate (NaOH 0.24 mol·L^−1^ and K_2_S_2_O_8_ 0.074 mol·L^−1^) [24,25]. Organic matter (OM) content was calculated according to the loss on ignition to constant mass (4 h) at 550 °C [26]. The pH of each sediment sample was measured in a 1:2.5 (*w/v*) mixture of sediment with deionized water [27]. The main properties of the collected sediments are shown in Table 1.

### 2.3. DNA Extraction

Sediment samples were stored at −80 °C until DNA extraction. DNA was extracted from 1 g fresh sediment samples by using a PowerSoil DNA Isolation Kit (Mobio Laboratories Inc., San Diego, CA, USA), following manufacturer protocol. DNA concentration and purity were checked by running the samples on 1.2% agarose gels. All the extracted total DNA samples were stored in −80 °C before further analysis.

### 2.4. PCR Amplification of 16S rRNA Genes and Sequencing

The V4-V5 regions of bacterial 16S rRNA genes were amplified using the universal primers 515F (GTGCCAGCMGCCGCGGTAA) and 926R (CCGTCAATTCMTTTRAGTTT), these primers were chosen because of their high coverage of almost all phyla in conventional and metagenomic studies [28,29,30]. The primers also contained the Illumina 5’ overhang adapter sequences for two-step amplicon library building, following manufacturer protocol for the overhang sequence. The amplification of bacteria gene fragments was achieved after two PCR steps.

PCR amplification was performed in a 25 μL reaction volume with 1 μL DNA template, 250 μM dNTPs, 0.25 μM of each primer, 1X reaction buffer, and 0.5 U Phusion DNA Polymerase (New England Biolabs, Ipswich, MA, USA). PCR cycling was carried out in a thermocycler under the following conditions: an initial denaturation at 94 °C for 2 min, followed by 25 cycles of denaturation at 94 °C for 30 s, annealing at 56 °C for 30 s and extension at 72 °C for 30 s, with a final extension of 72 °C for 5 min. The Illumina Nextera XT Index Kit (Illumina Inc., San Diego, CA, USA) with dual eight-base barcodes was used for multiplexing. Eight cycles of PCR reactions were used to incorporate two unique barcodes on both ends of the 16S amplicons. Cycling conditions consisted of one cycle at 94 °C for 3 min, followed by eight cycles of 94 °C for 30 s, 56 °C for 30 s, and 72 °C for 30 s. The final extension cycle was at 72 °C for 5 min.

Prior to library pooling, the barcoded PCR products were purified by using a DNA gel extraction kit (Axygen, Shanghai, China) and quantified by using the Qubit dsDNA HS Assay Kit (Life Technologies, New York, NY, USA). The libraries were sequenced by 2 × 300 bp paired-end sequencing on the MiSeq platform (Illumina, San Diego, CA, USA). Experiments were conducted at Tiny Gene Bio-Tech Co., Ltd. (Shanghai, China).

### 2.5. Statistical Analysis

The overlap between the forward and reverse reads was 180–190 bp approximately. The minimum quality score was 25 when merge the Illumina reads and the minimum overlap was 10 bp. The preprocessing of sequences was performed mainly by using MOTHUR 1.35.1 (University of Michigan, Ann Arbor, MI, USA) and by following the MiSeq analysis pipeline outlined in http://www.mothur.org/wiki/MiSeq_SOP [31]. Operational taxonomic units (OTUs) are defined as groups among which sequence similarities were greater than 97%. The species richness estimators (the abundance-based coverage estimator ACE, Jackknife and Chao1), Shannon diversity index, and Simpson diversity index were calculated. In this study, data preprocessing and OTU-based analysis were performed by MOTHUR (University of Michigan, Ann Arbor, MI, USA). Canoco 4.5 (Microcomputer Power, Ithaca, NY, USA) was used with Monte Carlo permutation test to perform the redundancy analysis (RDA) based on population abundance and environment factors. LEfSe (University of Auckland, Auckland, New Zealand) was used to find indicator bacterial groups specific to the sediment samples [32]. The statistical analysis was performed using SPSS 20.0 (International Business Machines Corporation, Armonk, NY, USA).

### 2.6. Accession Numbers

All of the sequencing data analyzed in the present study can be downloaded from the NCBI’s Sequence Read Archive using accession numbers SRR3354421 and SRR3354422.

## 3. Results and Discussion

### 3.1. Richness and Diversity of Microbial Community

**Figure 2:**
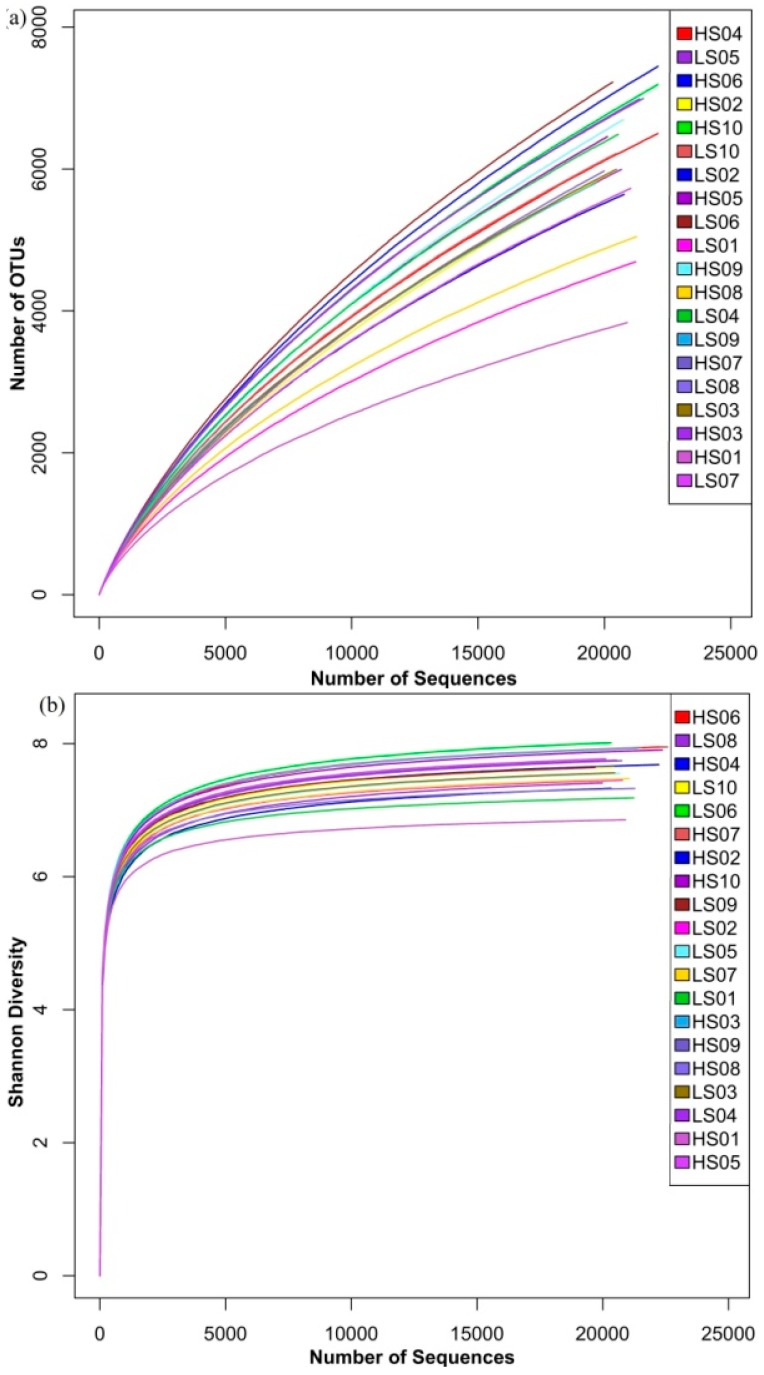
Rarefaction curves for (**a**) OTU (Operational taxonomic units); and (**b**) Shannon index were calculated using MOTHUR with reads for each sample using 0.03 distance OTUs.

The bacterial 16S rRNA gene sequences were obtained from sediment samples in different locations during the high and low water periods. Diversity concerns both taxon richness and evenness, and the results demonstrated that both parameters in most of sediments (HS3–HS10) from high water period were higher than sediments (LS3–LS10) from low water period (Figure 2). Tags with 97% similarity (Needleman–Wunsch alignment) were grouped according to OTUs to calculate the richness and diversity indices. The number of bacterial 16S rRNA gene sequences obtained from samples varied from 19,691 (LS9) to 22,586 (HS6). Table 2 shows the community richness and diversity by using different types of statistical methods, including ACE, Chao1, Simpson, and Shannon, and the end point of the curve (Figure 2) was used to calculate these indices. In comparing the diversity indices (Simpson and Shannon), the Shannon index showed that the sediment samples during the high water period (HS3–HS10) had a higher diversity than during the low water period (LS3–LS10), and the analysis of variance results indicated there were significant differences (*p* < 0.05) among the samples before and after impoundment. HS1 and HS2 samples had lower Shannon indices (6.85 and 7.33) and higher Simpson values (0.0041 and 0.0056, respectively) than LS1 and LS2. These two sampling positions were located nearby the estuary of the Yangtze River, and where different types of sediments from other places came together. Therefore, the slow flow caused the sedimentation of a large amount of sediments, which caused high diversity of the bacterial community during the low water period. Furthermore, with the exception of samples from S1, the sediment samples during the high water period (HS2–HS10) had a higher richness (Chao1 and ACE estimator) than during the low water period, and there were significant differences between the samples from high and low water periods (*p* < 0.05), thereby presenting a similar trend to diversity indices. HS6 also had the highest value of Chao1 and ACE (17,351 and 27,279, respectively).

### 3.2. Phylum Level Taxonomic Distribution

**Figure 3:**
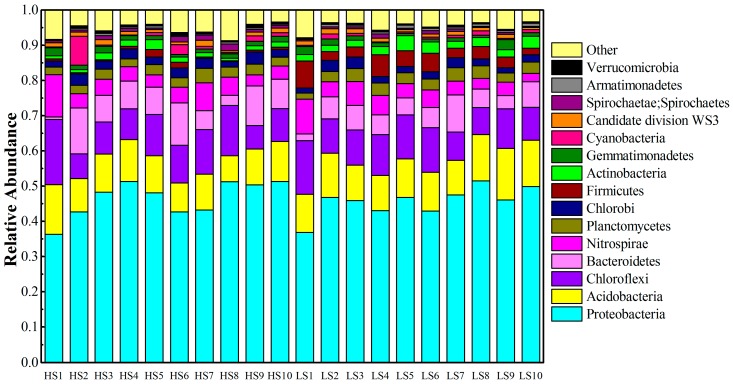
Phylogenetic distribution of sequences assigned on the phylum.

Among the filtered sequences, a total of 33 phyla were determined in the sediment samples. The dominant groups of each sample are displayed in Figure 3. *Proteobacteria* was the most abundant (36.4%–51.5%) phylum across all samples, and the sediment samples during the high water period had relatively higher abundance of *Proteobacteria* than those from the low water period. In additon, bacterial sequences were also affiliated with *Acidobacteria* (11.0% on average), *Chloroflexi* (10.9% on average), *Bacteroidetes* (6.7% on average), and *Nitrospirae* (5.1% on average). Sequences affiliated with *Firmicutes*, *Cyanobacteria*, *Planctomycetes*, and *Actinobacteria* were relatively abundant. The samples during the low water period had higher abundance of *Firmicutes* than the samples during the high water period, and the analysis of variance results indicated that there were significant differences (*p* < 0.01) among the samples before and after impoundment. HS1 and LS1 had higher abundance of *Nitrospirae* than samples from other points of collection. With the exception of samples from S1, S7, and S8, the abundance of *Nitrospirae* during the low water period was higher than during the high water period (*p* < 0.01). The relatively high abundance of *Cyanobacteria* (8.2% and 2.7%) was respectively observed in the samples of HS2 and HS6, and the samples of LS2 and LS6 had lower abundance (<2%) of *Cyanobacteria* during the low water period.

*Proteobacteria* is reportedly the most abundant phylum in soil and sediment [33,34]. In this study, the most abundant bacterial phylum was *Proteobacteria*. *Acidobacteria*, and *Chloroflexi*, which are common in lake sediments [33,35], and were also abundant in the present study. The HS1 and LS1 samples were located in east Dongting Lake. They had higher TN concentrations in interstitial water (monitoring data, 1.89 mg·L^−1^) and overlying water (monitoring data, 5.67 mg·L^−1^) and nitrification sediment might have occurred frequently at the sediment-water interface. Therefore, HS1 and LS1 had higher abundance of *Nitrospirae* than the other locations. *Firmicutes* produces spores that could resist dehydration and extreme environment conditions. Therefore, the samples during the low water period had higher *Firmicutes* abundance than samples during the high water period. HS2 and HS6 had a relatively high abundance of *Cyanobacteria* during the high water period. A previous study reported that *Cyanobacteria* was dominant in the water column [36]. S2 was located in the outlet of Dongting Lake and the estuary of the Yangtze River, whereas S6 was located in the river estuary into Dongting Lake. The sediments from these two positions had high TN and TP concentrations (Table 1), and these positions showed eutrophication and algal bloom during the high water period.

### 3.3. PCoA on OTU Level and RDA of Community Abundance on Dominant Bacterial Phyla

**Figure 4:**
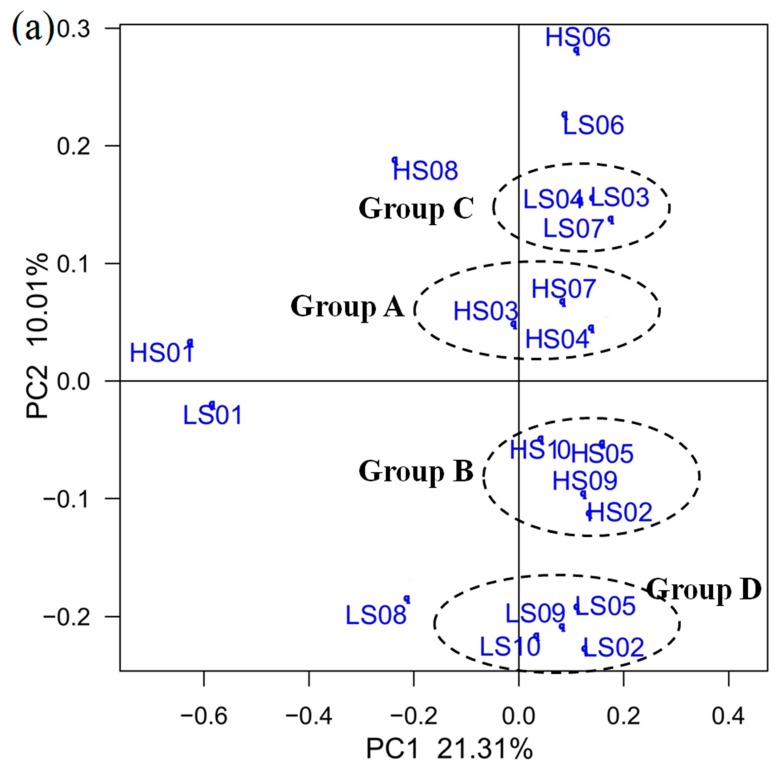

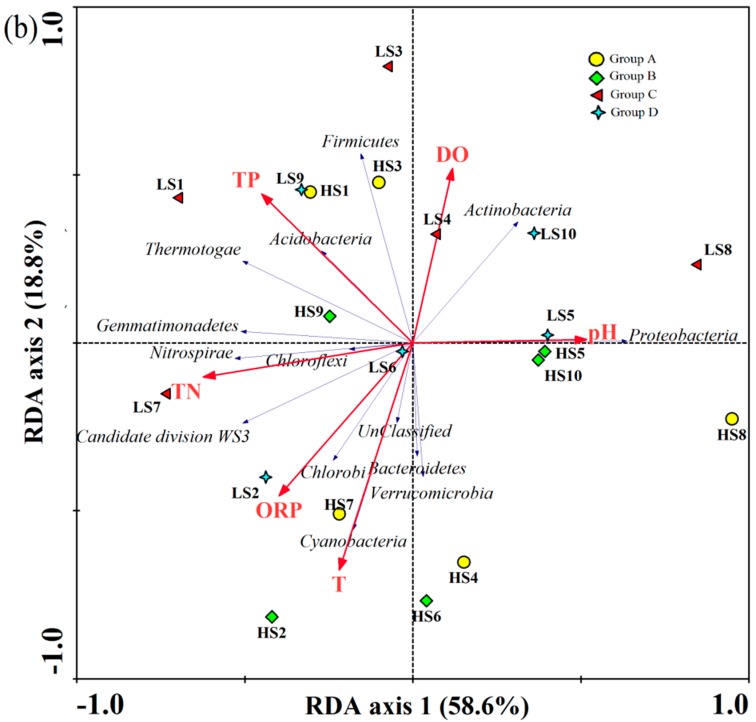
Ordination plot showing grouping of sediment samples according to their bacterial community structure (**a**); and the RDA (**b**) of the bacterial communities, as affected by environmental conditions, and based on the relative abundance of dominant bacterial phyla.

Principal coordinate analysis (PCoA) revealed differences in the patterns of the sediment bacterial community between samples from different locations and times on the OTU level. Figure 4a shows the grouping of sediment samples according to their bacterial community structure. First two PCoA axes explained 31.21% of the total variation on the microbial community structure. Bacterial communities are displayed clustering on the ordination plot according to sediment sample (Figure 4a), with the samples from the three lake districts of Dongting Lake during high and low water periods (Groups A and C), and the river estuary into Dongting Lake during high and low water periods (Groups B and D). Samples from S3, S4, S6 and S7 were gathered on the first quadrant, and HS6 or LS6 had lower similarity with the other samples from S3, S4 and S7 (Groups A and C). Samples, HS2, HS5, HS9 and HS10 (Group B), clustered on the fourth quadrant, as well as LS2, LS5, LS9 and LS10 (Group D). The analysis of significance test indicated that samples from high (Group A and B) and low (Groups C and D) water period had significant differences (*p* < 0.05).

RDA was performed based on population abundance and environment factors with Monte Carlo permutation test. Figure 4b shows the results after using sample-environment biplot. In Figure 4b, the first axis explained 58.6% of the bacterial diversity, whereas the second axis explained 18.8% of the variation. The results of Monte Carlo permutation test indicated that TN (*F* = 17.50, *p* = 0.014) and ORP (*F* = 12.33, *p* = 0.027) were significantly correlated with bacterial communities. pH exhibited negative relationships with TN, TP, ORP and T. DO was lowly correlated with the other environmental factors (*F* = 0.429). Changes in pH had a distinct effect on the community composition of samples from high (HS5, HS8 and HS10) and low water periods (LS5, LS8 and LS10) compared to the other samples. *Proteobacteria* was the most abundant and the largest phylum in sediment samples from S5 and S10, and pH might be related to its abundance [37]. Three factors (TN, ORP and T) were related to the community compositions of the other samples (HS2, LS2, HS7 and LS7). TP was related to the bacterial communities of the samples from S1, S3 and S9, and *Acidobacteria* was enriched in sediment samples (HS1, HS3, LS1 and LS3) from Groups A and C.

### 3.4. LEfSe Analysis Based on Community Abundance

LEfSe is an effective algorithm for high-dimensional biomarker discovery and for explaining detailed identification of abundance features, which characterizing the differences between two or more biological conditions [32]. This method was designed to analyze data, wherein the number of species is much higher than the number of samples and to provide biological class explanations to establish statistical significance, biological consistency, and effect-size estimation of predicted biomarkers [32]. Generally, this tool can analyze bacterial community data at any taxonomy level. In this study, we performed statistical analyses from phylum to genus levels.

**Figure 5:**
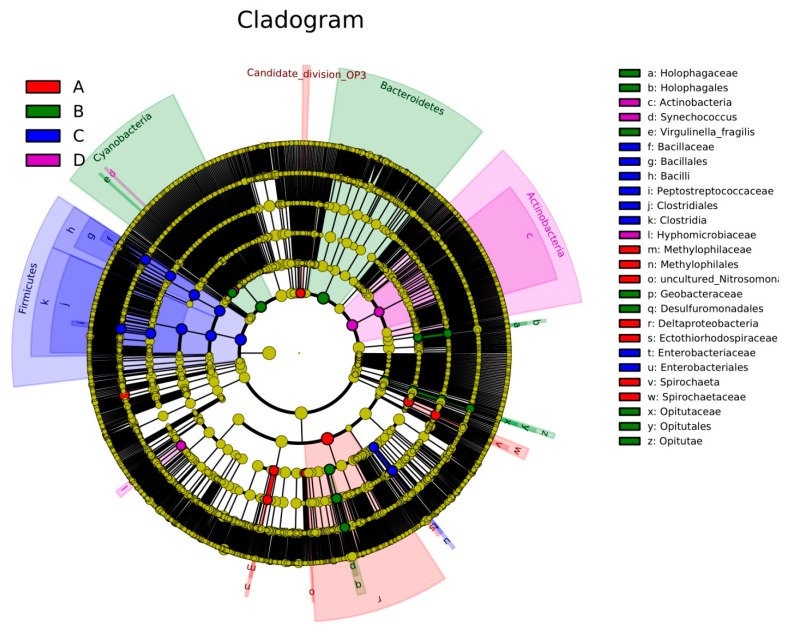
Cladogram indicates the phylogenetic distribution of microbial lineages associated with the four groups of sediments; lineages with LDA values of 2.0 or higher (as determined by LEfSe) are displayed. The black circles from inner to outer stand for phylum, class, order, family, genus, and species. Red, green, blue, and purple circles stand for the samples from Groups A, B, C, and D, respectively.

**Figure 6:**
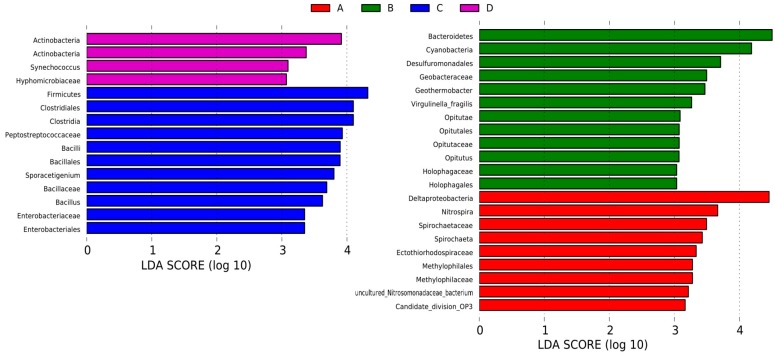
Indicator microbial groups within the four groups of sediments with LDA values higher than 3.0.

Cladogram (Figure 5) show taxa with the default logarithmic (LDA) values (Figure 6) higher than 3.0 for clarity. The bacterial lineages mainly enriched in Group A were *Candidate_division_OP3*, *Spirochaetaceae* (an order from *Spirochaetales*), *Deltaproteobacteria*, and *Methylophilales* (an order from *Betaproteobacteria*), as shown in Figure 5. Among these, only *Spirochaeta* and *Methylophilaceae* showed LDA values higher than 3 in Group A sediment (Figure 6).

Six groups of bacteria were abundant in Group B, namely *Cyanobacteria* (from phylum to class), *Bacteroidetes* (from phylum to genus), *Desulfuromonadales* (an order from *Deltaproteobacteria*), *Holophagales* (within *Acidobacteria*), *Opitutae* (within *Verrucomicrobia*), and *Chitinivorax* (within *Betaproteobacteria*), as shown in Figure 5. Within these groups, four fine lineages had an LDA value of 3 or higher, namely *Holophagaceae*, *Virgulinella_Fragilis*, *Geobacteraceae*, and *Opitutaceae* (Figure 6).

The *Firmicutes* and *Gammaproteobacteria* (the class and their order of *Enterobacteriales*) were enriched in the Group C, particularly the *Bacilli* and *Clostridia* in *Firmicutes* (Figure 5). Figure 6 also indicates that *Bacillaceae*, *Peptostreptococcaceae*, and *Enterobacteriaceae* had LDA values higher than 3.

Five groups of bacteria were present in Group D (see Figure 5), namely Firmicutes, Cyanobacteria, Actinobacteria, Nevskiaceae, and Shewanellaceae (within Gammaproteobacteria), and Hyphomicrobiaceae and Rhizobiaceae (within Alphaproteobacteria). Among these, only *Hyphomicrobiaceae* showed a LDA value higher than 3 among the Group D sediments (see Figure 6).

With the help of high sequence numbers, our study determined diverse lineages, and almost all common phyla were observed in each group of sediment. Within their phylum, *Betaproteobacteria* and *Deltaproteobacteria* were enriched in the sediment samples from Groups A and B, *Gammaproteobacteria* was enriched in the sediment samples from Groups C and D, and *Alphaproteobacteria* was enriched in the sediment samples from Group D. As the most diverse and even community, the samples from Groups B and D had the highest number of indicator taxa distributed in a variety of lineages, of which *Cyanobacteria* and *Actinobacteria* were the major indicators, respectively (Figure 5 and Figure 6). The samples from Groups B and D were collected from the river estuary into Dongting Lake, and diversity of bacterial community was high because of the complex environmental conditions. In the river estuary into Dongting Lake, the eutrophication was relatively steep during the high water period, and *Cyanobacteria* were enriched. *Actinobacteria* commonly existed in soil or sediment, particularly under the conditions with low water content or high organic matter [38]. Therefore, during the low water period, the samples from Group D were rich with *Actinobacteria*. The sediment samples from the three lake districts of Dongting Lake during the high water period (Group A) and low water period (Group C) were mainly enriched with *Deltaproteobacteria* and *Firmicutes*. In order to withstand the dehydration and extreme environment, a high amount of *Firmicutes* existed in sediments during the low water period. This might be the cause of high abundance of *Firmicutes* in Group C.

## 4. Conclusions

The sediment bacterial community in downstream linked lakes was greatly influenced by the operation of large-scale water conservancy projects. In Dongting Lake, the bacterial communities had significant differences before and after impoundment of the TGD. The results show that the sediment samples before impoundment of the TGD had higher community diversity and richness than after impoundment. The most abundant phylum obtained by the sequence affiliated to the bacterial domain is *Proteobacteria* in both water periods. *Betaproteobacteria* and *Deltaproteobacteria* were highly abundant in the sediment samples before impoundment of the TGD. The abundance of *Gammaproteobacteria* in the sediment samples after impoundment of the TGD was high. *Acidobacteria*, *Chloroflexi*, *Bacteroidetes*, and *Nitrospirae* were also relatively abundant phyla in the sediment samples. The TP and DO concentrations affected bacterial communities of the samples from S1 and S9. The bacterial communities of the samples from S2 and S7 were related to the TN, ORP concentrations, and the water temperature.

The sediment bacterial community in downstream linked lakes is important to the entire lake’s ecosystem. Understanding the profiling of sediment bacterial community in downstream linked lakes before and after impoundment of the water conservancy projects is crucial to lake preservation and control. Taking a large downstream linked lake, Dongting Lake, as the example, new insights into the bacterial communities in the sediments of Dongting Lake and valuable references for such communities before and after impoundment of the TGD are provided.

## Figures and Tables

**Table 1:** Physicochemical properties (mean value ± standard deviation) of the sediments.

Sample	TP (mg·kg^−1^)	TN (mg·kg^−1^)	Water Content (%)	OM (%)	pH
HS1	892.3 ± 10.2	620.2 ± 6.1	36.98 ± 2.13	8.21 ± 0.65	7.16 ± 0.44
HS2	982.2 ± 9.2	1184.2 ± 8.2	44.55 ± 3.21	8.1 ± 0.49	7.35 ± 0.48
HS3	722.4 ± 8.2	705.5 ± 8.1	47.31 ± 3.76	6.17 ± 0.46	7.25 ± 0.53
HS4	823.2 ± 8.5	1004.6 ± 8.9	63.02 ± 4.29	7.62 ± 0.56	7.17 ± 0.49
HS5	892.8 ± 8.6	992.1 ± 8.5	40.36 ± 5.75	5.97 ± 0.48	7.42 ± 0.61
HS6	1003.7 ± 9.1	1197.8 ± 12.4	43.51 ± 3.98	7.07 ± 0.49	7.29 ± 0.67
HS7	698.2 ± 7.3	821.2 ± 10.2	46.02 ± 3.57	5.16 ± 0.51	7.27 ± 0.62
HS8	782.3 ± 7.8	995.3 ± 8.3	48.84 ± 3.43	7.03 ± 0.56	7.36 ± 0.68
HS9	892.3 ± 7.7	1045.1 ± 10.3	35.11 ± 3.01	6.68 ± 0.41	7.46 ± 0.61
HS10	602.3 ± 7.2	611.9 ± 8.3	30.76 ± 2.79	6.41 ± 0.62	7.37 ± 0.56
LS1	921.2 ± 8.2	842.3 ± 7.3	33.21 ± 2.76	8.52 ± 0.69	7.21 ± 0.49
LS2	1021.4 ± 9.2	1302.3 ± 8.1	40.12 ± 3.65	8.32 ± 0.72	7.43 ± 0.48
LS3	823.1 ± 8.5	923.1 ± 9.4	44.21 ± 3.96	7.01 ± 0.67	7.31 ± 0.42
LS4	937.5 ± 9.3	1198.5 ± 12.3	52.13 ± 4.02	7.34 ± 0.78	7.21 ± 0.51
LS5	983.3 ± 9.1	1123.2 ± 12.8	36.21 ± 4.23	6.12 ± 0.51	7.39 ± 0.63
LS6	1193.1 ± 10.2	1423.2 ± 11.3	38.97 ± 4.47	7.56 ± 0.68	7.33 ± 0.71
LS7	821.5 ± 7.8	983.5 ± 11.7	40.46 ± 4.21	6.05 ± 0.59	7.35 ± 0.58
LS8	842.1 ± 7.2	1045.2 ± 10.8	42.36 ± 3.76	7.12 ± 0.69	7.36 ± 0.62
LS9	982.4 ± 8.7	1197.3 ± 11.5	30.21 ± 3.28	7.24 ± 0.66	7.41 ± 0.51
LS10	701.2 ± 8.8	801.6 ± 10.3	27.98 ± 2.67	6.89 ± 0.57	7.51 ± 0.57

**Table 2:** Diversity of the 16S rRNA gene libraries from the sequencing analysis.

Samples	Total Reads	OTU	Chao1	ACE	Simpson	Shannon
HS1	20,903	3836	7863	10,727	0.0041	6.85
LS1	21,231	4685	9569	13,925	0.0027	7.19
HS2	20,323	5968	14,318	24,153	0.0056	7.33
LS2	20,777	5631	11,931	17,402	0.0025	7.45
HS3	21,389	6977	15,218	22,448	0.0012	7.92
LS3	20,475	5964	13,828	21,097	0.0021	7.55
HS4	22,239	6569	14,308	22,036	0.0018	7.75
LS4	20,546	6511	14,138	21,108	0.0017	7.69
HS5	20,118	6509	15,405	23,734	0.0016	7.77
LS5	20,668	6048	13,522	20,455	0.0021	7.56
HS6	22,586	7534	17,351	27,279	0.0014	8.12
LS6	20,328	7193	16,320	25,817	0.0011	8.01
HS7	21,547	7000	15,147	22,413	0.0012	7.93
LS7	21,042	5709	12,116	18,138	0.0021	7.47
HS8	21,271	5047	14,636	23,396	0.0022	7.48
LS8	19,989	5963	9953	14,672	0.0031	7.32
HS9	20,751	6670	16,138	25,942	0.0017	7.74
LS9	19,691	5750	12,903	20,436	0.0016	7.63
HS10	22,367	7233	16,542	25,599	0.0013	7.90
LS10	20,452	6159	13,562	20,807	0.0020	7.64
